# Virtual reality-assisted assessment of paranoid ideation in forensic psychiatric inpatients: A mixed-methods pilot study

**DOI:** 10.3389/fpsyg.2023.1242243

**Published:** 2023-12-07

**Authors:** Richard Hedström, Märta Wallinius, Kristina Sygel, Chris N. W. Geraets

**Affiliations:** ^1^Research and Development Unit, Regional Forensic Psychiatric Clinic, Växjö, Sweden; ^2^Evidence-Based Forensic Psychiatry, Department of Clinical Sciences Lund, Psychiatry, Lund University, Lund, Sweden; ^3^Department of Psychiatry and Neurochemistry, Center for Ethics, Law and Mental Health, Institute of Neuroscience and Physiology, The Sahlgrenska Academy at University of Gothenburg, Gothenburg, Sweden; ^4^Department of Forensic Psychiatry, National Board of Forensic Medicine, Stockholm, Sweden; ^5^Department of Psychiatry, University Medical Center Groningen, Groningen, Netherlands

**Keywords:** virtual reality, assessment, diagnostics, paranoia, psychiatry, mental disorders, forensic psychiatry

## Abstract

**Background:**

Reliable and valid assessment of paranoia is important in forensic psychiatry for providing adequate care. VR technology may add to current assessment procedures, as it enables observation within realistic (social) situations resembling the complexity of everyday life. VR constitutes a promising tool within forensics, due to the restricted nature of forensic psychiatric hospitals and ethical challenges arising from observing potentially dangerous behaviors in real life.

**Objective:**

To investigate the feasibility of VR assessment for paranoid ideation in forensic psychiatric inpatients qualitatively by assessing the experiences of patients and a clinician, and to explore how the VR measures relate to established clinical measures.

**Methods:**

One clinician (experienced psychiatrist) and 10 forensic psychiatric inpatients with a history or suspicion of paranoid ideation were included. Patients participated in two immersive VR scenarios (bus and supermarket) during which paranoia was assessed by the clinician. Qualitative interviews were performed with patients and the clinician performing the assessment to investigate experiences and feasibility. Further, measures of paranoia, social anxiety, and positive symptoms were obtained.

**Results:**

Nine out of 10 participants with varying levels of paranoid ideation completed the assessment. Manifest inductive content analyses of the interviews revealed general experiences, advantages such as enabling observing participants from a different perspective, and challenges of the VR assessment, such as a lack of objectivity and the laboriousness of the assessment for the clinician. Although more paranoia was experienced during the supermarket scenario, correlates with classical measures were only significant for the bus scenario.

**Discussion:**

The VR assessment was appreciated by most patients and the clinician. Based on our results short, standardized VR assessment scenarios are feasible, however, they do not appear reliable or objective for assessing paranoia. The clinical usefulness is most likely as a collaborative tool and add-on measure to existing methods.

## 1 Introduction

Paranoid ideation is one of the most common symptoms of a psychotic disorder (Freeman, 2007). Such thoughts can be mild or severe and manifest as persecutory delusions, which are characterized by the belief that harm is occurring, or will occur, and that someone intends to inflict harm (Freeman, 2007). Up to 90% of patients with a psychotic disorder experience paranoid ideation to some degree (Moutoussis et al., 2007). In forensic psychiatry, where patients with a combination of severe mental disorders and criminal conduct are treated, paranoid ideation is prevalent and has been proposed as an underlying, and sometimes even causal, risk factor for violent offenses (Coid et al., 2016; Darrell-Berry et al., 2016).

Reliable and valid assessment of paranoia is important for providing adequate healthcare for patients with psychotic disorders and may in some cases also be relevant to the formulation of violence-preventive strategies for patients. Current assessments in forensic psychiatry mainly consist of clinical interviews, self-reports, and staff observations (Aboraya et al., 2005). A downside of self-reports and interviews is that they rely on memory, insight, and motivation of patients. Furthermore, the secluded environment strongly differs from life outside the clinic, which can affect the reliability and ecological validity of observations. Novel technologies such as virtual reality (VR) could potentially provide new possibilities for psychiatric assessments (Freeman et al., 2017; van Bennekom et al., 2017; Geraets et al., 2022).

Immersive VR enables patients to interact with computer-generated virtual worlds, usually by wearing a head-mounted display. VR simulations have been shown to trigger emotional, psychological, and physical reactions similar to real-life reactions (Martens et al., 2019). To effectively elicit such emotions and responses, VR simulations have to induce a sense of “presence.” For a VR user to experience presence requires experiencing a sense of both “place illusion” and “plausibility illusion”; that is believability of both the virtual environment itself and the unfolding scenario (Slater, 2009; Skarbez et al., 2017; Geraets et al., 2021).

Several systematic reviews have investigated the existing evidence on VR assessment for paranoia (Freeman et al., 2017; van Bennekom et al., 2017; Rus-Calafell et al., 2018). An advantage of VR for the assessment of paranoia lies in its controllability. In a VR environment, we can manipulate, and thus know, whether virtual characters (avatars) show friendly, neutral or hostile behavior. In contrast, when using self-report of daily life situations, it is unknown whether the self-reported paranoia or hostility reflects a persecutory delusion or whether it reflects an actual, imminent social threat. As such, in a VR situation with exclusively neutral cues, a patient’s self-reported paranoid ideation and level of perceived hostility are easier to evaluate. Among the reviewed VR paradigms are scenarios involving riding the underground with several avatars, and exploring public environments such as cafés, a library, and a supermarket, while informing on thoughts of participants about the virtual scenarios directly afterward (Rus-Calafell et al., 2018). The reviews conclude that almost all reviewed VR scenarios could elicit and measure self-reported paranoia in clinical and non-clinical populations to some extent (Freeman et al., 2017; van Bennekom et al., 2017; Rus-Calafell et al., 2018). However, not all studies agree that VR can reliably differentiate between clinical and non-clinical groups. In accordance with this, many of the current studies have been performed as proof-of-concept studies for VR environments or to investigate mechanisms. A lack of knowledge still exists concerning the clinical use and potential for VR-assisted assessment of paranoid ideations.

Virtual reality technology may add value to current forensic psychiatric assessment procedures, as it enables observation within realistic (social) situations resembling the complexity of everyday life, where cognition and behavior can be monitored in real time (Riva, 1997; Freeman et al., 2017; Pan and Hamilton, 2018). Further, VR has the advantage that you can (repeatedly) expose people to (social) situations that are controlled, safe, and can be accessed within the isolated high-security environment of the clinic. VR has been used safely in both patients with paranoia and forensic psychiatric patients (e.g., Pot-Kolder et al., 2018; Klein Tuente et al., 2020). Thus, the use of VR in assessments constitutes a promising tool especially within forensic psychiatry, considering the restricted nature of forensic psychiatric hospitals and the ethical challenges arising from observing potentially dangerous behaviors in real-life settings (Freeman et al., 2017; van Bennekom et al., 2017).

In the current pilot study, we investigated a novel VR-assisted assessment for paranoid ideation in forensic psychiatric inpatients. This was done both qualitatively and quantitatively by (1) evaluating how the VR-assisted assessment was experienced by patients and the clinician, (2) investigating how clinicians’ observations from VR scenarios can add to paranoia assessments, and (3) describing how the VR measures relate to established clinical measures of paranoia and anxiety.

## 2 Materials and methods

### 2.1 Design and participants

In total, 10 forensic psychiatric inpatients from a high-security forensic psychiatric clinic in Sweden were included in this single-group, mixed-methods pilot study. Inclusion criteria were: (1) aged 18 or older, (2) currently receiving forensic psychiatric treatment, and (3) a history of, or indications of current, paranoid ideation. Exclusion criteria were: (1) insufficient command of the Swedish language, (2) inability to provide informed consent due to current psychiatric status, (3) presence of an organic brain disease, e.g., dementia or epilepsy, and (4) posing severe security risks (e.g., violence) hindering safe participation. A clinician was recruited, from the consultant psychiatrists at the clinic, to perform the VR-assisted assessments.

Both quantitative and qualitative data were gathered from the patients through interviews, self-reports, observations, and medical record reviews. Qualitative (interview) data was obtained from the clinician conducting the VR-assisted assessment.

### 2.2 Ethics

This study was approved by the Swedish Ethics Review Authority (2021-06353-01) and was conducted according to the principles of the Declaration of Helsinki. Treating psychiatrists only referred patients to the study if they were assessed as able to provide informed consent and did not pose current and severe security risks. The patients were informed, verbally and in writing, about the study by a research assistant. Specific care was taken to explain that participation or non-participation would not in any way affect the patients’ inpatient care and that they could cease participation at any time without providing a reason. If patients were willing to participate, written, informed consent was obtained.

### 2.3 Procedures

Patients were referred to the study by their treating psychiatrist, according to inclusion and exclusion criteria. After eligible participants had been informed of the study, those who wanted to participate signed informed consent. Subsequently, background data were collected, and the participants completed the VR scenarios and measurements. The clinician could discontinue a VR scenario when there was a risk of harm (e.g., due to falling). Participation took between 1.5 and 4 h and could be completed in 1 or 2 days (in case it was too intensive to finish all the measures on the same day). Four participants completed all measures within the same day. Five patients completed the clinical trait measures (see the measurement section) the next day, and one patient finished it 2 days later. During the VR scenarios and VR-specific measures, both a clinician and a research assistant were present. After completion of participation, participants received a gift voucher of 99 SEK (approx. $9) for the kiosk at the clinic.

### 2.4 VR scenarios

**FIGURE 1:**
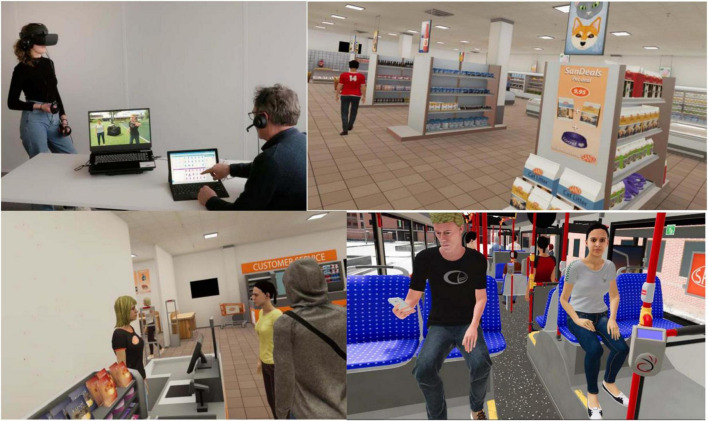
The VR setup and the VR environments as seen by the patients in Scenarios 1 and 2. Reproduced with permission from CleVR.

In this study, the Social Worlds^©^ VR software created by CleVR (Delft, The Netherlands) was used. Participants used an Oculus Rift S to view the virtual environments, wore headphones, and moved around using Oculus controllers, see Figure 1. The clinician guided the participant through the VR scenarios by using a microphone. First, a 2-min VR practice scenario was performed, and then a supermarket and bus scenario each lasting 5 min. These two virtual environments were chosen as these are neutral environments and relatable to most people, even for those who have been incarcerated or involuntarily confined to a hospital for a long period of time.

**Practice scenario:** The participant was immersed in a virtual supermarket and was free to explore the supermarket to become accustomed to the VR experience and equipment, and to report signs of cybersickness. The virtual environment was programmed to include 12 freely moving customer avatars, which meant a moderate level of crowding where participants passed avatars throughout the supermarket, but never were surrounded by them. Avatars would shortly turn their attention to participants only if they came close. No other interaction with avatars was possible and the avatars were set to continuously show a neutral facial expression toward the participant.

**Scenario 1:** Directly after the practice scenario, the participant was instructed through the headphones to search the virtual supermarket for milk and to inform the clinician once he or she had found it. Next, the participant was instructed to find the cashier and to queue behind the avatars until the scenario ended after 5 min. Due to the random movements of the 12 avatars (acting neutrally) participants faced different numbers of avatars while queuing, and some participants experienced avatars “cutting the line” from behind. During this scenario only ambient sounds were present and avatars, including the cashier, would not respond to any potential comments by participants.

**Scenario 2:** The participant was seated on a bus which left the bus station and drove through a city neighborhood. On the bus were 11 other avatars, all programmed to act neutrally. The participant could not move around the environment but could look around the bus in 360 degrees as it moved. A female and a male avatar were seated opposite the participant. During the bus ride, the participant overheard the female avatar (enacted by the clinician using the microphone with voice distortion) having a (scripted) heated telephone conversation about her being late (“Mhm. I’ve told you I will be late! Mhm. No!” etc.). The male avatar would occasionally look at the participant, pick up his phone or look across at the female avatar at moments when she spoke louder. After ending the call, the female avatar turned to the participant and asked for directions in a calm and neutral manner. The scenario ended after the participant answered.

### 2.5 Quantitative measurements

#### 2.5.1 Sociodemographic characteristics

Age, gender, diagnoses, and IQ scores were collected from patients’ medical records. IQ scores were measured with the Wechsler Adult Intelligence Scale Version R, III, or IV. Reliability for the WAIS-R has only been estimated for its subscales, which were deemed moderate to good (ranging from 0.65 to 0.88) (Wechsler, 1996). Full-scale reliability for the later versions were deemed excellent: 0.90 for WAIS-III (Wechsler and Nyman, 2003) and 0.96 for WAIS-IV (Wechsler and Nyman, 2010).

#### 2.5.2 Clinical trait measures

Positive psychotic symptoms were assessed using the Positive and Negative Syndrome Scale interview (PANSS). The Positive symptom subscale is made up of seven items measuring the presence and severity of positive symptoms on a 7-point Likert scale. This subscale has good test-retest reliability of α = 0.81 and interrater-reliability of 0.73 (Kay et al., 1989).

Social anxiety was measured with the Social Interaction Anxiety Scale (SIAS). The SIAS consists of 20 items assessing the tendency to fear and avoid social situations on a scale from 0 (not at all) to 4 (extreme), resulting in a total score ranging between 0-80. Internal and test-retest reliability for the original English version is considered very good, while showing discriminant validity toward non-clinical samples, depression, and other anxiety disorders (Mattick and Clarke, 1998).

Paranoid thoughts were assessed with the Revised Green Paranoid Thoughts Scale (R-GPTS). The R-GPTS has two subscales: Ideas of Reference (8 items) and Ideas of Persecution (10 items). Items are rated on a 5-point Likert scale, ranging from 0 (not at all) to 4 (totally), the total score ranges from 0 to 72. The instrument is considered to have excellent reliability, especially at elevated levels of paranoia (Freeman et al., 2021).

#### 2.5.3 VR measures

State anxiety levels were measured before and directly after each VR scenario on a verbal analog scale (VAS) by rating current anxiety on a scale from 0 (not at all) to 100 (extremely anxious).

VAS perceived hostility was assessed after each VR scenario by asking: “How hostile were the people in the VR environment toward you, on a scale from 0 (not hostile) to 100 (extremely hostile).”

State paranoia in each VR scenario was assessed with the 20-item State Social Paranoia Scale (SSPS). Ten items assess state paranoia, i.e., negative intention about the virtual characters (e.g., “Someone was hostile toward me”) and 10 items describe positive or neutral interpretations of the virtual characters. Items were rated on a 5-point Likert scale, resulting in scores ranging from 10 to 50 on state paranoia. The original English instrument has demonstrated excellent internal reliability and adequate test-retest reliability. Combined with clear divergent and convergent validity it is considered to have good psychometric properties (Freeman et al., 2007).

Presence in VR was assessed after completing both scenarios using the 14-item Igroup Presence Questionnaire (IPQ). Items were scored on a 7-point Likert scale and analyzed according to previously established factors of general presence, spatial presence, involvement, and realness. The instrument has demonstrated good psychometric properties (Schubert et al., 2001).

### 2.6 Qualitative data

During the scenarios, the participant’s behavior in VR (physical and verbal expressions) was observed by the clinician. Behavior was rated using a structured observation protocol with open questions assessing (1) social physical behavior (e.g., “Does the participant look at avatars, does he avoid avatars, etc.”), (2) emotional expressions (“What emotions does the patient show during the VR session? How?”), (3) verbal expressions (“Does the patient say anything? What?”), and (4) other observations considered relevant for the assessment of symptoms of paranoia, see Supplementary Table 1 for the observation protocol. The clinician’s observations of the participant’s physical and verbal expressions in VR were summarized and manually divided into categories.

The clinician conducted a semi-structured interview with the participant regarding his/her experiences in VR directly after the scenarios, to gain more insights into symptom-related experiences in VR. The interviews were audio recorded and transcribed using the NVivo software.

To assess the acceptability and feasibility of the VR simulation, the researcher conducted semi-structured interviews (see Supplementary Table 2) with both the participant and the clinician on their experiences with the VR assessment. All interviews were audio recorded and transcribed using the NVivo software.

### 2.7 Data analyses

Descriptive statistics were calculated for all quantitative data by presenting the mean and standard deviation or n, and the median and range. Explorative, non-parametric Spearman’s rho correlation analyses were performed to assess relations between VR state paranoia measures (SSPS and VAS scores) and the clinical trait measures (PANSS, R-GPTS, and SIAS). Significance was accepted at 0.05 due to the explorative design of the study. For visualization of VR state and trait measures spider graphs were made per participants, by transforming the scores of each measure to percentages.

Analysis of the qualitative data was conducted through manifest inductive content analysis using NVivo, in accordance with the process described by Vears and Gillam (2022). The interview scripts were coded by authors RH and MW and then summarized into content categories and subcategories using an iterative process.

## 3 Results

**FIGURE 2:**
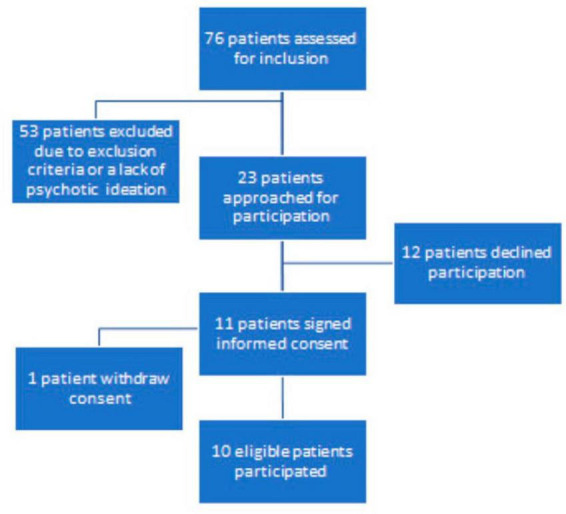
Visualization of the selection and recruitment process.

Between March 2022 and May 2022 23 inpatients from the clinic’s high-security units, and 53 inpatients from the clinic’s medium security units were assessed for inclusion with their treating psychiatrist (see Figure 2). Exclusion occurred mainly due to not having a history of paranoid ideation or current symptoms, a cognitively impaired psychiatric state, lacking Swedish language skills and/or severe risks of violence. The risk of violence was most pronounced for high-security candidates, but was also a factor when excluding several patients in medium security. Out of the original 76 patients, 23 medium security inpatients fulfilled the criteria and were approached for the study. In total, 11 patients signed informed consent. One patient subsequently withdrew consent, resulting in a final sample of 10 participants: a 43% inclusion rate. Table 1 shows the demographic and clinical characteristics of the participants: 9 out of 10 had a current diagnosis of a psychotic disorder. Participants represented diverse psychiatric treatment histories, with a wide range of psychotic experiences and length of outpatient and inpatient care.

**TABLE 1:** Participant characteristics and clinical measures (*n* = 10).

	M (SD) or N	Median	Range
**General characteristics**			
Age in years	35.8 (9.6)	33.0	29–62
Female	2		
Male	8		
IQ estimate	9		
Within 85–115 range	7		
Within 85–95 range	2		
Number of diagnoses	2.1 (0.7)	2.0	1–4
Minimal one comorbidity	9		
Years since first reported psychotic symptoms	8.3 (3.3)	9.5	3–12
Years of forensic inpatient care	6.3 (10.4)	2.2	0.3–35
**ICD-10 diagnoses**			
Schizophrenia and Other Psychotic Disorders	9		
Substance-Abuse Disorders	7		
Autism and Other Pervasive Developmental Disorders	1		
Attention-Deficit Disorders	1		
Personality Disorders	2		
**Clinical measures**			
Positive symptoms (PANSS)	13.0 (5.8)	10	7–23
Delusions	2.7 (2.2)	1.5	1–6
Suspiciousness	3.4 (2.2)	2.5	1–7
Conceptual Disorganization	1.8 (1.0)	1.5	1–4
Hallucinatory Behavior	1.4 (1.3)	1	1–5
Excitement	1.1 (0.3)	1	1–2
Grandiosity	1.1 (0.3)	1	1–2
Hostility	1.5 (0.7)	1	1–3
Paranoid thoughts total (R-GPTS)	21.6 (20.8)	21	0–61
Ideas of social reference (R-GPTS A)	8.8 (9.8)	6	0–31
Average (0–9)	7		
Elevated (10–15)	1		
Moderately severe (16–20)	1		
Severe (21–24)	0		
Very severe (>24)	1		
Ideas of persecution (R-GPTS B)	12.8 (15.8)	5	0–46
Average (0–4)	4		
Elevated (5–10)	2		
Moderately severe (11–17)	0		
Severe (18–27)	3		
Very severe (>27)	1		
Social interaction anxiety (SIAS)	27.9 (22.0)	28	2–75

**FIGURE 3:**
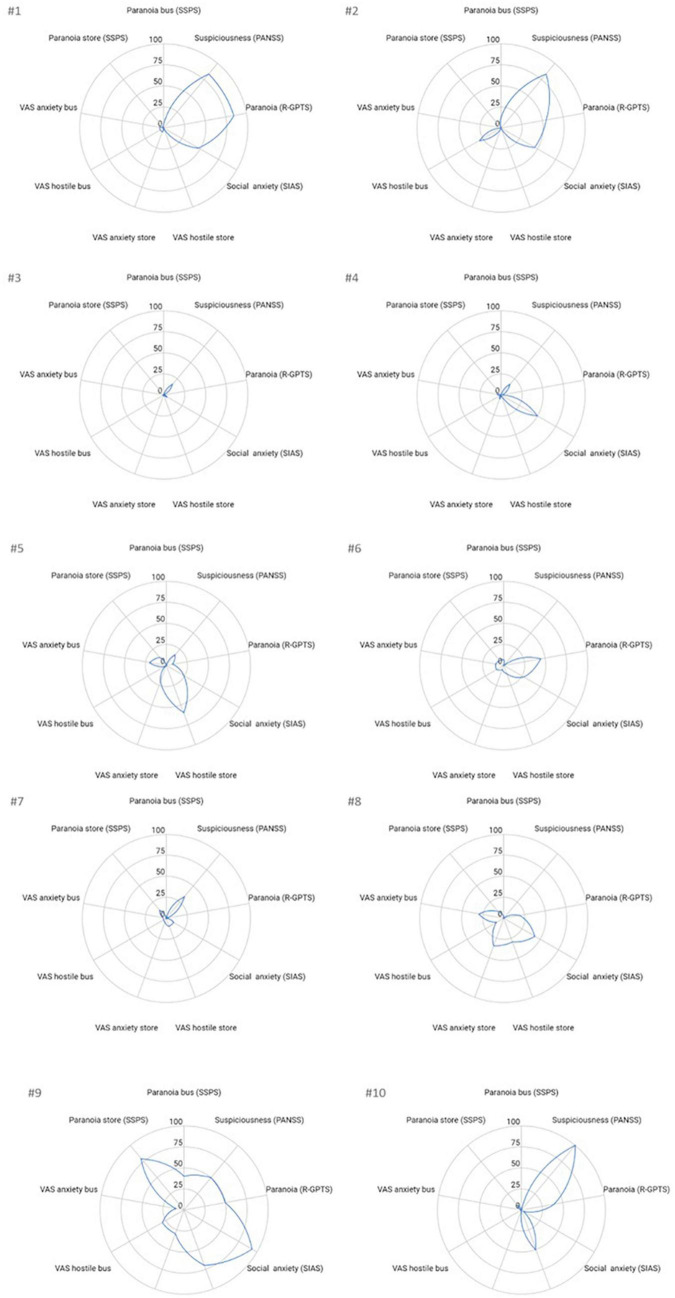
Visualization of trait and VR state measures per participant. The standardized data of participants #1 to #10 is provided for paranoia and anxiety related traits (PANSS, R-GPTS, and SIAS) and state (VAS scores and SPSS) measures. To enable comparison each score was transformed into percentages by dividing the score by the maximum score possible of that measure and multiplying by 100.

The sample showed a diverse presentation of current positive psychotic symptoms as measured with the PANSS. Among the 10 participants, the most noteworthy symptoms were suspiciousness/persecution (*n* = 1 extreme case, *n* = 2 severe cases) and delusions (*n* = 2 severe cases, *n* = 1 moderately severe case). Absent or minimal positive psychotic symptoms were found among three participants. Regarding paranoid ideation as measured in R-GPTS, one participant showed very severe ideas of social references, while four participants showed severe or very severe ideas of persecution, respectively (see Table 1 and Figure 3).

### 3.1 Experiences of VR-assisted assessment by the patients and the clinician

Nine participants completed both VR scenarios. One participant only completed the bus scenario, as the clinician discontinued the supermarket scenario (after ± 3 min) after observing balance issues. Two other participants reported minor symptoms of cybersickness and showed varying degrees of balance issues during the supermarket scenario, but could continue. One participant asked to be seated during the supermarket scenario, due to fear of standing while wearing the VR-glasses. No serious adverse events were reported.

Participants experienced moderate presence in VR on all four subscales of the IPQ (range 0–6): general *M* = 5.1 (SD = 1.0) spatial presence *M* = 4.0 (SD = 0.9), involvement *M* = 3.4, (SD = 1.1), and realness *M* = 2.9 (SD = 1.1). Table 2 presents the manifest inductive content analyses of the interviews with the patients and the clinician regarding their perceptions of the VR-assisted paranoia assessment. In total, three content categories with specific subcategories were identified: (1) advantages of VR in assessment, (2) experiences with VR assessment, and (3) challenges administering VR scenarios.

**TABLE 2:** Manifest inductive content analysis with illustrative quotes from interviews with participants and clinician.

Explanation	Quote
Content category 1. Advantages of VR in assessment	
**1.1. Appreciation of the VR-mediated assessment format**	
VR scenarios were experienced as easy to participate in, even by patients previously expressing fear of using VR, with several preferring the VR task to standard clinical interviews/questionnaires. The clinician saw VR scenarios as a potential add-on to gold-standard interviews, as the VR context makes it possible to interact with/observe patients in a role other than as a clinical interviewer.	“It was better like this (…). Because when I talk to people, then they will not understand me. (…) But then, when they do understand, they get so scared that I have to change hospitals!” *(#10*) “Yes, some of them actually showed greater capacities or (…) an interest to collaborate than I expected. (…) There were some other sides to them, at least. I’m thinking of (participant). Now, he showed completely different sides to me! Well, maybe as a doctor I haven’t seen these sides, because… At the ward and in the psychiatrist role (…) you have certain limitations in your contacts with patients. But there were other things in VR.” (C)
**1.2. Information gained by VR assessment**	
Participants interpreted avatars’ behaviors as both negative (e.g., “rude,” “irritated,” and “unsympathetic”) and positive. Many patients experienced one or several avatars as hostile or condescending, based on eye contact or other cues (e.g., avatars approaching too closely, avatars being too quiet/too loud). The clinician emphasized how VR scenarios made it possible to observe patients from a third-person perspective, which was particularly helpful with patients who also communicated openly about their social interpretations in the VR context. This provided an increased understanding of paranoid interpretations and social skills, especially for patients with limited social experiences due to long periods of inpatient care and/or interpersonal difficulties.	“- It wasn’t possible to… It’s hard to know, actually. Because if he would have been standing there I would have asked him.” (#5) - If you had the option of talking to him (…), what would you have said? (C) - I would probably have asked why… Why he’s not standing in the queue? So, why is he standing there, staring? But that could provoke him too! Then maybe it’s better not to say anything? (…) So, you better not start something unnecessary neither. (…) But you better keep in the back of your head that it’s good to stay vigilant, yes…” (#5) “We do see that (patients) have difficulties, but maybe it’s a little (…) clearer in VR? Because sometimes it is hard for them to tell us, or they do not want to discuss their symptoms (…). It can be of help to assess them in these situations. (…) Yes, there were actually at least two for whom it seemed that there was a lot, an awful lot, going on below the surface. Even if we suspect that all is not entirely well. But, the description that patients give about their conditions, or what we observe in the wards is not enough, and that’s where a few other things show specifically during VR. Now I am thinking about a few patients regarding psychotic symptoms and one of them regarding communication overall, and how this person feels in social situations.” (C)
**Content category 2. Experiences with VR assessment**	
**2.1. Attitudes to VR technology**	
Many participants expressed interest and curiosity for the VR technology, either because of its novelty, associations with video games or, in one case, because of the clinic’s existing VR treatments. However, a few participants described simultaneous worries and fears about VR technology, e.g., fears of provoking or worsening psychotic symptoms, and a general uneasiness with the technology. The clinician expressed concern for one participant’s strong fear of VR, as well as the possibility of handling patients’ possible fears adequately within the 1-1.5-h timeframe of the experiment.	“It was really amazing. You know, this 360-degree vision you get, seeing everything around you instead of watching things on a screen!” *(#4)* “It felt like you almost needed to follow up, because (VR) provoked so many (…) worries and thoughts… You have to be prepared for… Really, some thoughts and symptoms can come to the foreground and actually need to be addressed later on, after the session.” (C)
**2.2. Perceived realness of VR**	
Many participants described, or acted in accordance with, feelings of being immersed and present in the VR experience. These feelings were linked to the perception that avatars were actively looking at or interacting with them. However, some described VR as “different” and “virtual,” or as “feeling unreal,” linking it to awareness of the outside world, physical sensations from the VR equipment, limitations set by the VR scenarios (e.g., being unable to interact with objects and avatars or to move freely), and the design and behaviors of avatars which made several participants perceive them as odd, incomprehensible, non-human or “programmed.” The clinician experienced the scenarios as unrealistic because of the scripted nature and wished for more interactions and freedom to navigate and try problem-solving in the VR setting.	“Yeah, no, but it’s more that I can’t actually imagine personalities and feelings and a consciousness in those characters. So that made it really difficult to answer these questions. So, if you mean… It is sort of a question of definitions for me. Noticed me? Well, then I guess, sure, she was talking to me so she did notice me, even if it didn’t happen.” *(# 4)* “And of course you can stand in a queue when nothing is happening, but then that will be something different… Either there is no cashier, in which case you have to resolve that? For example, what if you are in a real situation in a store, then you’ll have to think, like: What do I do now? Should I leave and… Or something along those lines. Should I go look for the cashier? Should I wait until someone else does it? So there you have lots of scenarios.“ (C)
**2.3. Patient engagement**	
During follow-up interviews, all participants at times gave neutral or brief answers, with little or no self-reported suspiciousness about avatars, also when answering open-ended questions. The majority described most avatars as ordinary and unremarkable, or as lacking discernable emotions. The clinician observed varying levels of participant engagement between and during the scenarios and the follow-up interviews, with length of inpatient treatment as a possible confounder.	“- But what did you think of the avatars, those characters? (C) - Nothing special. (#3) - What were they doing? (C) - They just sat there, talking on the phone. (#3) - What kind of people were they? (C) - They were regular people? (#3) - How did they make you feel? (C) - Nothing special (#3) - What were they thinking about you? (C) - They didn’t show” (#3) “Some of (the participants), maybe (…) the ones who haven’t been inpatients for such a long time, maybe it’s not as exciting for them to do something they haven’t… Could it be that they are not as motivated as the ones for whom nothing happens, and now something does?” (C)
**Category 3. Challenges administering VR scenarios**	
**3.1. The role of randomness and chance in VR scenarios**	
The clinician noted that participants’ decisions on how to explore and navigate the scenarios influenced their experiences, e.g., where, when, and how they saw or encountered avatars. Further, avatars also moved randomly, resulting in variance in avatars noticing, looking at, and approaching participants. This affected the participants’ experiences, and made them non-comparable between participants.	“That guy behind me was awfully close. Although, I went… (…) It’s hard to say. I don’t think they. Honestly, I reacted to them and got kind of alert and careful back there.” *(#5)* “Yes, I do understand that this is specific software, but in practice there will be enormous differences for the patients. Some take an endless amount of time to find products in the store and then they barely queue, while others who moved faster or just found their way by chance, had to wait for a real long time in this queue, which doesn’t really exist (as avatars do not respect the queue, they do not take out groceries). And then, this is obviously not a natural situation.” (C)
**3.2. Patients’ social experiences in relation to VR scenarios**	
Participants described being with new people (in VR) as an unfamiliar experience, which for some made a strong impression. Simply being talked to or being in a social situation with avatars of the opposite sex was, for some, an unusual experience. The clinician described how participants’ varying social experiences, as well as length of inpatient care, seemed to affect their social skills and comfort in social interactions, specifically at the supermarket or public transport environments. Accordingly, some participants described the VR as “enjoyable,” while others brought up lifelong difficulties and uneasiness in stores and public transport.	“It was a little bit scary right there when you entered the store and then, all of a sudden, there were people everywhere and… That experience was some kind of smaller shock, you could say. Especially when you have been isolated from a lot of people yourself, that… But that passed pretty fast. My first thought was just that it must have been a long time since I found myself in a store.” *(# 4)* “The comments from the patients. (…) That someone appreciated (…) just to have another person look at them (…). I would not have guessed that it was so important for this patient to be paid attention to in that way. And then it was interesting to observe how paranoid someone gets just by queueing in the grocery store, for example. So, it really felt like that person was terribly afraid back there… And another interesting piece of information was seeing how some of our patients… How long ago it was that they were among people outside the clinic (…) and how stressful those situations can be, like riding the bus or visiting a grocery store, even though it’s VR.” (C)
**3.3. Misunderstandings in scenarios and interviews**	
In follow-up interviews, some participants struggled noticeably with understanding interview questions - several expressed difficulties answering or asked for rephrasing. Also, some participants gave tangential answers, possibly related to psychotic thought processes. The clinician noticed that some participants misunderstood or were confused by scenarios. This was partly attributed to a lack of clear tasks in the experimental design, partly to distortions in voice transformation and partly to misunderstandings due to psychotic symptoms. Especially brief answers by participants made their understanding hard to evaluate.	“(The avatars) were just busy with their phones, right? There was someone who asked a question, but I didn’t know if that was directed to me or to someone else.” *(#6)* “These details (…) It really should be… I don’t know, practiced or seen to somehow beforehand, in order to give the best results. So that it feels natural and not that… That the participants do not understand who is talking to them. Is it me or the avatars?” (C)
**3.4. Usability and fit of VR equipment**	
Some participants experienced problems with the size of the VR glasses, or discomfort using the glasses. Several participants either expressed or were observed by the clinician as having difficulties navigating in the VR context, with concerns regarding standing up or bumping into objects/avatars. The clinician described feeling stressed and discouraged when using the equipment and occasionally needing to restart the software, and recommended thorough software training and practice for clinicians.	“Yes, the weight of the VR glasses also kept me reminded that all this is not real. I would want them to weigh less, that’s my opinion. When you move your head, then, then that actually felt a bit heavy.” *(#6)* “In my opinion, there are technical things that need to be massively improved and planned for, before we start using this at a bigger scale. Because now the technical side was actually what influenced the most and didn’t always make it possible to (…) work without issues. That’s what popped up all the time, you know?” (C)
**3.5. Simultaneous use of VR equipment and clinical observations**	
Administering the protocol required simultaneously running the VR scenarios, observing and documenting behaviors. The clinician experienced this as too complex, with too many simultaneous tasks, and recommended future administrations to be conducted in teams of two clinicians.	“The tricky part was… Well, there were several aspects. (…) It was planned from the beginning that I was supposed to run the program and observe the patient at the same time. That means I have to watch the screen and observe the patient at the same time, which in my opinion has been a challenging task. Because on the one hand I have to observe how they interact with the avatars (…) on the screen, where they direct their attention and where they’re going and so on. (…) But at the same time, I can also observe the patient’s mimics and speech and verbal reactions. And so that they don’t get dizzy (…). For me this was absolutely impossible!” (C)

### 3.2 Clinician observations during VR

Using the structured observation protocol (Supplementary Table 1), the clinician mainly noted participants’ direction of gaze and movement patterns, see Table 3. Interpretations of interactions were made in four cases, and in three cases participants gave verbal information on how they interpreted the avatars’ behaviors or actions and recorded observations of emotions with positive,

**TABLE 3:** Summary of clinician’s observations according to type of behavior/reaction.

Types of observation and number of participants in which the observation occurred	Illustrative examples: Supermarket scenario//Bus scenario
**Topic 1. Social behaviors**	
• Direction of gaze, e.g., at environment, at avatars by gender (*n* = 10) • Patterns of movement, e.g., hesitant, active, walk into avatar (*n* = 5) • Patterns of interaction, e.g., cautious, inquisitive, hesitant, none (*n* = 4)	• Hesitant toward avatars in the queue//looks more at male (#5) • Almost walks into some//looks at both (#2) • Active, inquisitive, walks into avatars//looking at both, especially male (#6) • //barely shifts gaze from avatars (#9)
**Topic 2. Emotional pressure**	
• Emotions with positive valence, relaxation, curiosity, interest (*n* = 3) • Emotions with neutral valence, e.g., “neutral,” “no emotions” (*n* = 7) • Emotions with negative valence, i.e., anxiety, impatience (*n* = 5) • Description of participants actions, e.g., “snorts,” pacing, looking around” (*n* = 5)	• No emotions//interest, looking around (#3) • Nothing apparent//no apparent emotions (#7) • Restless, difficulty standing still//impatient, restless (#9) • Pacing//no emotions, impatient, shaking leg (#1)
**Topic 3. Verbal statements**	
• Seeking further verbal instruction, e.g., asks how to move, if avatar is speaking (*n* = 4) • Narrating and/or explain their experience, e.g., repeats instructions, nothing is happening, expressing opinion on program, mentions hostile gaze of avatars, explains hostility score, afraid to stand up (*n* = 9) • Misunderstanding scenario (*n* = 2) • Observing emotional valence of communication with avatars, e.g., friendliness, politeness, derision (*n* = 2)	• Asks how to move//misunderstands then communicates with avatar (#7) • Repeats instructions//asks if female is taking to him (#5) • Reporting and wondering about next step//no (#4) • Comments on cutting in but shows patience//friendly and polite toward speaker (#10)
**Topic 4. Other**	
• Additional information, e.g., speed, gait, and misunderstandings of the task (*n* = 4)	• Solves task quickly//(#9)

neutral, or negative valence. Furthermore, several participants narrated their experiences during the session. In two cases, the clinician noted interpretations of the emotional valence of the participants’ communication with the avatars. Furthermore, the clinician noted that two patients did not fully experience the bus scenario as anticipated, which was observed as they did not listen to and/or answer the female avatar as expected; one answered the female character as she was on the phone, and both were unsure whether the female avatar’s final question was directed at them.

### 3.3 Associations between VR assessments and clinical measures of paranoia and anxiety

Means, standard deviations, medians, and ranges of the VR assessment measures are presented in Table 4. Both VR paranoia measures, the SSPS state paranoia, and the single-item VAS hostility measure, correlated highly for both the supermarket (*r* = 0.90, *p* < 0.001) and the bus scenario (*r* = 0.94, *p* < 0.0001). On average, slightly more anxiety and paranoia were elicited by the supermarket scenario than the bus scenario. This is also reflected in the visualizations of the standardized scores of the state VR measures and clinical trait measures for each participant in Figure 3, where a strong heterogeneity between the profiles of patients can be noted. Some participants only showed elevated scores on established trait measures (#1, #2, #10) and not on VR measures, while some showed almost no paranoia or anxiety symptoms on any of the measures (#3, #7). Others (#8, #9) demonstrated a more integrated picture with elevated scores on both trait and VR measures. One participant (#5) reported a high level of experienced hostility in the supermarket scenario, but low scores on all other measures.

**TABLE 4:** VR measures.

	M (SD) or N	Median	Range
VR-specific measures			
State paranoia supermarket (SSPS)	15.2 (9.6)	13	10–42
State paranoia bus (SSPS)	12.7 (5.0)	10.5	10–26
VAS anxiety pre	10.0 (18.5)	3	1–60
VAS anxiety supermarket	10.9 (12.7)	5.3	1–35
VAS anxiety bus	8.7 (9.5)	5	1–30
VAS hostility supermarket	23.3 (27.2)	10	1–70
VAS hostility bus	9.3 (11.5)	4.5	1–30

#### 3.3.1 Supermarket scenario

Although the supermarket scenario triggered slightly more paranoid ideas, state paranoia (SPSS) and VAS hostility scores did not correlate with any of the clinical trait measures significantly. Also, for VAS anxiety scores, no clinical trait measures correlated. This indicates that people higher in trait anxiety and paranoia were not more prone to feeling anxious or paranoid during the VR exposures.

#### 3.3.2 Bus scenario

State paranoia (SSPS) in the VR bus scenario correlated strongly with social interaction anxiety (*r* = 0.64, *p* = 0.05), ideas of social reference (*r* = 0.77, *p* < 0.01), ideas of persecution (*r* = 0.66, *p* = 0.04) and the paranoid thoughts total score (*r* = 0.81, *p* < 0.01), but not with PANSS delusions or suspiciousness. Similarly, VR VAS hostility during the bus scenario correlated strongly with ideas of social reference (*r* = 0.69, *p* = 0.03), and the total score on paranoid thoughts (*r* = 0.67, *p* = 0.04), but not with social interaction anxiety or PANSS delusions and suspicious. Thus in contrast to the supermarket, significant associations were found, however, when interpreting it should be noted that people scored rather low on state paranoia and anxiety measures for this scenario.

## 4 Discussion

The present study is, to our knowledge, the first to examine the feasibility and clinical relevance of VR-assisted assessment of paranoid ideations in a clinical forensic psychiatric setting. The ten participants showed a wide range in severity of psychotic symptoms. Overall, many were positive and curious about the assessment, even though hesitations and fears of the VR technology and its possible effects emerged. From the clinician’s view, VR enabled observations of patients from a third-person perspective, and to initiate conversations on paranoia that otherwise would be difficult to create preconditions for. Several challenges were identified, such as difficulties in the practical use of VR while simultaneously performing clinical assessments and a lack of objectivity due to amongst others, variance in the avatar’s automated behavior.

Self-reports of paranoid ideations in VR were partially related to trait paranoid ideation and social anxiety, but a lack of associations to the clinically assessed PANSS scales, was demonstrated.

### 4.1 VR-assisted assessment as perceived by patients and clinician

Many participants described VR scenarios as a novel and interesting experience when compared to standard clinical interviews. Several participants who initially worried about the VR technology and its possible impact on their wellbeing, still preferred VR over standard interviews. Interestingly, worries about VR, slight discomfort from equipment or side effects (cybersickness) did not make patients drop out of the experiment. The only discontinued participant was initiated by the clinician, as there was a risk of falling due to balance issues in VR which were clearly noticeable to the clinician but described by the participant as minor cybersickness. Although postural instability has been reported in other VR research (Tian et al., 2022), several studies with forensic and paranoid patients have not reported high rates of cybersickness or balance problems (Rus-Calafell et al., 2018; Klein Tuente et al., 2020). The balance issues could be related to the participants’ unfamiliarity with VR, or the single-session format in which three scenarios were performed in a short period of time. Adaptations such as placing someone in a seated position may solve this issue.

Regarding two of the most central features of VR – experiences of presence and immersion – participants had diverse experiences. Some described experiencing strong presence and immersion, while some described circumstances affecting presence and immersion negatively, e.g., the feeling of wearing the equipment, restrictions in possible actions with the VR environment, and avatars’ “unnatural” expressions and movements. Thus, several participants described experiencing breaks in presence that could be classed as breaks in either place illusion, plausibility illusion, or co-presence (Slater, 2009). Conversely, others reported a continued sense of presence and strong emotions, even when for example bumping into avatars or not being answered when talking to avatars in the supermarket scenario. More investigations of factors affecting presence and immersion are needed.

The clinician appreciated the VR assessment as an alternative means of communication with patients, underlining how VR facilitated observations and conversations with the participants who were most suspicious or hostile to forensic psychiatry and clinical interviews. This is in line with previous studies, indicating that VR interventions potentially increase the degree of personal disclosure in other psychiatric settings (Pan and Hamilton, 2018). We therefore, humbly, suggest that VR may constitute a way to establish constructive two-way communication with patients who, due to hostility, severe paranoia, or previous negative experiences from standard interventions in compulsory (forensic) psychiatric care have been less responsive to previous interventions. Further clinical advantages with VR were described by the clinician as a means for roleplay and observations for patients with limited exposure to social situations, either because of long-term inpatient care or more generalized social difficulties.

The scenarios created for the current study were short and designed to be both neutral and standardized, while letting participants approach avatars and explore the surroundings. This design still contained variations between administrations, and follow-up interviews revealed that the scenarios themselves also carried different meanings for different participants. For example, avatars moving randomly and participants exploring freely in the supermarket scenario meant that participants could bump into avatars and avatars rush past participants, causing different experiences and social situations. Thus, the ability to interact with VR environments, while potentially contributing to maintaining the plausibility illusion of a VR scenario, also creates more variation in the assessment scenario, thereby limiting standardization and objectivity (Pan and Hamilton, 2018). Furthermore, interviews revealed that the scenarios related differently to patients’ specific psychotic symptoms, cognitions, and social learning histories. A wide range of attitudes and reactions were reported to the supposedly neutral scenario environments, ranging from excitement about a virtual environment outside the clinic to VR environments triggering anxieties. These differences must be taken into consideration when evaluating our results since these experiences could be one explanation for why the three most paranoid participants did not report paranoid ideations during VR.

A prominent challenge for the clinician was the limited feasibility of simultaneously conducting clinical assessments while managing the VR technology. We acknowledge that this could be mitigated through automated VR scenarios, or through having an assistant performing the VR scenarios, allowing for the clinician to completely focus on observations of patients’ behaviors. However, when protocols are less strict and not focused on objective assessment, this might also release strain on the clinician, as has been observed in treatment studies where conducting interactive scenarios and providing feedback (thus observing) with similar software was feasible for clinicians (Nijman et al., 2022). Also, providing more thorough training for the clinician (than was done for the current study) with the VR hardware and software seems to be an important feature if such assessments are to be implemented in clinical practice.

### 4.2 Paranoia in VR and associations with standard assessments

Paranoia in the VR scenarios was reported by some participants and was partly observed by the clinician. The clinician’s observations showed a range of reactions to the scenarios (e.g., curiosity, hesitancy, lack of interest), but no strong signs of paranoid ideations and behavior among participants. For the majority of those demonstrating paranoia during the VR scenarios, the supermarket scenario provoked more paranoia than the bus scenario on both the single-item VAS scale and the state paranoia questionnaire. Inspection of the graphs demonstrating the overall paranoid symptoms presented by each participant showed that those presenting high scores at PANSS and R-GPTS scales, in general, did not experience anxiety or paranoia to a large degree during the VR scenarios. When associations were investigated in correlation analyses, no significant associations between VR-related measures of state paranoia and the clinically assessed PANSS scales were demonstrated, such relations were only found for self-reports of social anxiety and paranoid ideations (in the bus scenario). However, it also should be noted that VR state paranoia measures do not necessarily need to match the interview and questionnaire-based scores, as they concern different timeframes. I.e., the VR measure concerns the past 5 min, whereas the R-GPTS concerns the past month, and the PANSS the past week. In previous experimental studies, participants in VR scenarios have expressed ideas of persecution, social evaluative concerns, risk of physical harm and emphasized social cues in both patients and non-clinical people with high trait paranoia (e.g., being approached too closely, being the subject of the avatars’ attention) (Freeman et al., 2008; Riches et al., 2020).

Given the obvious limitations of a very small sample, our results must be seen as preliminary. However, it provides information for future research that VR-elicited paranoia should be investigated in relation to clinically assessed paranoia, with no assumptions on the inherent overlap. Also, differences between clinical and non-clinical populations should be investigated, as a part of a validation process and investigating the utility as a potential “objective” assessment form, as small and non-significant differences have been reported previously when assessing paranoia during a VR underground train ride (Fornells-Ambrojo et al., 2015; Veling et al., 2016), though the majority of research did find significant differences between such groups (Rus-Calafell et al., 2018).

To be noted, two participants self-reported paranoid ideations in R-GPTS without receiving an elevated score of paranoia in the clinical PANSS interview. This could have been due to current real stressors and conflicts in the forensic psychiatric environment for these specific participants (i.e., actual threats), which was captured as paranoia by the R-GPTS. Fornells-Ambrojo et al. (2015) have previously highlighted the role of environment and adverse life-events in creating and maintaining paranoid ideations, recommending VR assessment to consider participants’ social background and different neighborhoods. For our sample, these aspects were arguably important, and we recommend continued VR research in forensic settings to take the social environments of patients into consideration.

### 4.3 Therapeutic misconceptions

Expectations reflecting therapeutic misconceptions were evident during recruitment, administration, and follow-up interviews. Therapeutic misconception denotes the misunderstanding and conflation of research goals, protocols, and procedures with clinical treatment effects (Thong et al., 2016). While many participants seemed to understand the study, three out of ten expressed both hopes and fears of VR as an “objective” measure of their mental health, even after thorough information on the study aims and procedures was provided by the research assistant. These participants either disagreed with their current diagnosis and wished for it to be reexamined through this study, or rather worried about showing potential early signs of new psychotic episodes that would be detected through VR. Referring psychiatrists expressed similar expectations about paranoid ideations being discernable through VR scenarios, despite the study repeatedly being presented as a pilot project without the possibility of diagnosing psychotic symptoms, and that no individual findings would be reported back to the referring psychiatrist. These misconceptions of the diagnostic capabilities of VR were comparable to therapeutic misconceptions encountered in psychiatric treatment studies, e.g., potential benefits from the study for the participant’s care, and misconceptions concerning the purpose of the study (Appelbaum et al., 2012). Our study design contained several elements previously found to increase the risk of therapeutic misconception, e.g., researchers having simultaneous clinical assignments and conducting the study in forensic psychiatric settings (Pedersen et al., 2021). The current sample is also characterized by established risk factors for therapeutic misconception in psychosis patients: residential living, poor independence in activities of daily functioning, cognitive deficits, and positive psychotic symptoms (Thong et al., 2016). The expectations and fears that were voiced in our study underline the importance of clear communication adjusted to the participants’ responsiveness, to decrease the risk of therapeutic misconception during research.

### 4.4 Limitations

The present study has several limitations. Firstly, 57% of the approached patients were not willing to participate, and we have no information on how these patients may have differed from those who chose to participate. Thus, the actual representativeness of the sample is unknown. However, this participation rate is comparable to other studies in forensic psychiatry (Pedersen et al., 2021). Second, participants’ IQ scores were collected through medical records, but measured at different points in time during their illness and cannot be assumed to be reliable measures of current intellectual functioning. Because of recruitment criteria, our sample may have fewer cognitive impairments when compared to patients in similar units and with similar lengths of stay, limiting the generalizability. However, the nature of this study, being an explorative feasibility study, does not entail strict considerations regarding representativeness and generalizability.

Further, the order of the two scenarios was not randomized and therefore we cannot rule out that this might have influenced the assessment and account for differences between assessments from the supermarket and bus scenarios. Also, we do not know whether the (minor) symptoms of cybersickness may have influenced results, a larger sample is needed to investigate this. Finally, the validity of self-report measures on paranoia poses additional limitations in forensic settings, where some individuals may face actual, physical danger in the forensic hospital environment (e.g., due to threats from fellow inpatients).

## 5 Conclusion

In this study, VR-assisted assessment of paranoid ideations proved overall acceptable to forensic psychiatric patients with different presentations of psychotic symptoms, social experiences, familiarity with VR technology and attitudes to their diagnosis. The VR format was appreciated by a subgroup of patients and the clinician, although the VR assessment (both the clinical model and the practical use of VR technology while conducting clinical assessments) should be revised to enhance practicality. Standardized VR assessment scenarios seem feasible to perform, however, the current research shows that they do not appear reliable as a stand-alone, objective assessment of paranoid ideations in forensic psychiatric patients.

Even though our VR assessment model did not identify clearly defined paranoid symptoms, there seems to be value in introducing VR-assisted assessment to forensic psychiatric practices. Moving forward, VR-assisted assessment could be examined as a collaborative and personalized tool, which could be especially relevant for patients who are hard to engage through standard methods. Accordingly, we recommend future research on personalized VR assessment scenarios for forensic psychiatric patients. Such scenarios could, for instance, examine more therapeutic-oriented goals instead of aiming for objective symptom measurements. Finally, the forensic psychiatric setting added additional challenges concerning expectations of VR technology, patients’ preoccupations with diagnoses in inpatient care, as well as the very real security concerns patients may face, all of which should be examined in future studies.

## Data availability statement

The raw data supporting the conclusions of this article will be made available by the authors, without undue reservation.

## Ethics statement

This study involving humans was approved by the Swedish Ethical Review Authority Box 2110 750 02 Uppsala. The studies were conducted in accordance with the local legislation and institutional requirements. The participants provided their written informed consent to participate in this study. Written informed consent was obtained from the individual(s) for the publication of any identifiable images or data included in this article.

## Author contributions

CG and MW designed the study. RH and CG wrote the first draft of the manuscript and carried out the quantitative analysis of the results. RH and MW analyzed the qualitative interview data. KS analyzed observation protocols from the VR scenarios. All authors contributed to manuscript revision and read and approved the submitted version.
