# Supplementary material for: Virtual reality-assisted assessment of paranoid ideation in forensic psychiatric inpatients: A mixed-methods pilot study
Source: Front Psychol. 2023 Dec 7;14:1242243. doi: 10.3389/fpsyg.2023.1242243 (PMC10733482; doi:10.3389/fpsyg.2023.1242243)
Supplement: Supplementary file 1 [file Table_1.pdf]

Supplement 1. Structured observation protocol for behavior in VR

Observe the patient's behaviors, reactions, emotional expressions and statements during the VR scenario. Pay special attention to the areas described below.

Type of behaviour/reaction	Examples of observations	Clinician's observations
Social behaviors	Is the patient approaching/avoiding avatars? Does the patient look at avatars? Which?	
Emotional pressure	What emotions does the patient show during the VR session? In what way?	
Statements	Does the patient say anything? What?	
Other	Other observations during the VR scenario deemed relevant for the assessment of paranoid psychosis symptoms	
