# Supplementary material for: Virtual reality-assisted assessment of paranoid ideation in forensic psychiatric inpatients: A mixed-methods pilot study
Source: Front Psychol. 2023 Dec 7;14:1242243. doi: 10.3389/fpsyg.2023.1242243 (PMC10733482; doi:10.3389/fpsyg.2023.1242243)
Supplement: Supplementary file 2 [file Table_2.pdf]

Supplement 2. Semi-structured interview with the clinician and researcher

Clinician-led interview with patient after VR session

1. What happened after you put on your VR glasses?

2. What did you think of the avatars?

3. What did they do?

4. What kind of people were they?

5. How did they make you feel?

6. What did they think of you?

7. Check the SSPS form on the previous page - follow up the item with high scores (4 or 5) with follow-up questions.

For example: *You filled in that different people were hostile, what did they do?*

Researcher-led interview with patient after VR session

Global experiences

1. How did you experience using VR?
2. What was it like to participate in an assessment with VR?
3. How did you feel using VR glasses?

Please rate these statements from 0 to 6:

1. Even when the others were present I felt alone in the VR-world.

    0 Do not agree
    6 Agree completely

2. I felt the presence of someone else in the room

    0 Do not agree
    6 Agree completely

3. I felt that the others noticed my presence in the VR-world

    0 Do not agree
    6 Agree completely

Follow-up questions on co-presence:

1. What else can you add to this?
2. Why did you rate this item this high/low?
